# Modelling determinants, impact, and space–time risk of age-specific mortality in rural South Africa: integrating methods to enhance policy relevance

**DOI:** 10.3402/gha.v6i0.19239

**Published:** 2013-01-24

**Authors:** Benn Sartorius

**Affiliations:** School of Public Health, Faculty of Health Sciences, University of the Witwatersrand, Johannesburg, South Africa

**Keywords:** mortality, space–time risk, determinants, population attributable fractions, demographic surveillance system, rural, South Africa

## Abstract

**Background:**

There is a lack of reliable data in developing countries to inform policy and optimise resource allocation. Health and socio-demographic surveillance sites (HDSS) have the potential to address this gap. Mortality levels and trends have previously been documented in rural South Africa. However, complex space–time clustering of mortality, determinants, and their impact has not been fully examined.

**Objectives:**

To integrate advanced methods enhance the understanding of the dynamics of mortality in space–time, to identify mortality risk factors and population attributable impact, to relate disparities in risk factor distributions to spatial mortality risk, and thus, to improve policy planning and resource allocation.

**Methods:**

Agincourt HDSS supplied data for the period 1992–2008. Advanced spatial techniques were used to identify significant age-specific mortality ‘hotspots’ in space–time. Multivariable Bayesian models were used to assess the effects of the most significant covariates on mortality. Disparities in risk factor profiles in identified hotspots were assessed.

**Results:**

Increasing HIV-related mortality and a subsequent decrease possibly attributable to antiretroviral therapy introduction are evident in this rural population. Distinct space–time clustering and variation (even in a small geographic area) of mortality were observed. Several known and novel risk factors were identified, and population impact was quantified. Significant differences in the risk factor profiles of the identified ‘hotspots’ included ethnicity; maternal, partner, and household deaths; household head demographics; migrancy; education; and poverty.

**Conclusions:**

A complex interaction of highly attributable multilevel factors continues to demonstrate differential space–time influences on mortality risk (especially for HIV). High-risk households and villages displayed differential risk factor profiles. This integrated approach could prove valuable to decision makers. Tailored interventions for specific child and adult high-risk mortality areas are needed, such as preventing vertical transmission, ensuring maternal survival, and improving water and sanitation infrastructure. This framework can be applied in other settings within the region.

Increased HIV infection and the re-emergence of tuberculosis (TB), especially in sub-Saharan Africa, has been accompanied by a resurgence of drug-resistant strains of malaria and TB closely associated with HIV/AIDS (1, 2). This has had a substantial impact on mortality in the region.

Reliable mortality data are a prerequisite for planning health interventions, yet such data are often not available or reliable in developing countries, including those in sub-Saharan Africa (3). In many instances, health and socio-demographic surveillance systems (HDSS), though not representative at the national level, are often the only means to assess and more clearly understand population levels, trends, and determinants on a prospective basis (4, 5).

Recent advances in data availability and analytic methods have created new opportunities to improve the analysis and modelling of diseases on a local, national, or regional basis (6, 7). Spatial analysis and, for example, Bayesian geostatistical modelling are powerful and statistically robust tools for identifying high-mortality areas in a heterogeneous and imperfectly known environment and associated determinants (6, 8). An increasing body of literature on spatial analysis of health outcomes in developing countries has been motivated by the availability of geo-referenced data and by the recent advances in methods and software that can implement such complex models (7, 9). The identification of geographical clusters of high-risk mortality is an important policy issue that has received limited attention, especially the ability to identify individuals, households, and villages at elevated risk. This study contributes to other literature that investigates mortality and its risk factors that are important from a public health perspective (10). The study also provides guidance regarding the distribution of health services and other spatially-targeted interventions for disease control, mortality reduction, and resource allocation in rural South Africa and has application to broader sub-Saharan Africa.

Addressing health inequities in populations is a major challenge (11), and research that documents and quantifies inequities is needed to inform policies to close health gaps in the developing world. Evidence on reducing inequities within countries is growing. Successful approaches include those that improve geographic access to health interventions in poor communities, subsidize health care and health inputs for the poor, and empower poorer communities (12).

This study aims to describe and develop a framework that captures the space–time dynamics and determinants of age-specific mortality in rural South Africa.

## Methods

### Study area and population

**Fig. 1:**
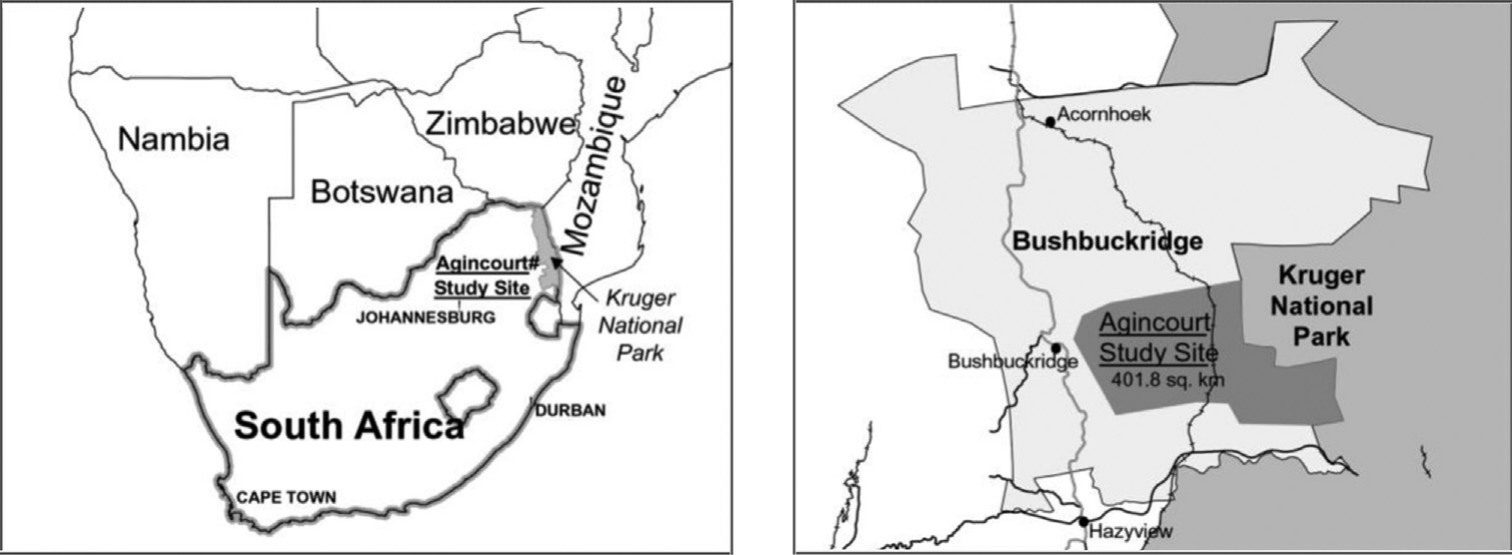
Maps showing the location of the Agincourt HDSS (41).

The Agincourt HDSS is located in a sub-district in north-east South Africa (Fig. 1). There was a baseline census in 1992 that collected data on all individuals and households in the population (13). This has been followed by annual updates of births, deaths, and in- and out-migrations. It is a poor rural sub-district that includes former Mozambican refugees, temporary migrant workers, and a more stable permanent population (13). The site at present covers an area of about 400 km^2^ and contains 25 villages, 13,500 households, and 84,000 individuals. There is a full geographic information system (GIS), containing locations of all households within the site, which is updated annually. A household is defined as a group of people who reside and eat together, plus the linked temporary migrants who would eat with them on return. Verbal autopsies (VAs), a method of determining individuals’ causes of death in populations without a complete vital registration system, were introduced in 1993. A full VA is conducted on every death recorded during the annual census update and is administered to the closest caregiver of the deceased by a trained fieldworker (14). Three medical practitioners assess VAs to determine likely cause of death. Causes of death (main, immediate, and/or contributing) are coded to be consistent with the International Classification of Diseases (ICD-10). The main cause of death was used in these analyses.

The study population comprised infants (<1 year), children (1–4 years), young adults (15–49 years), and older adults (50–64 years) in the original 21 villages for the period 1992–2008. Children aged 5–14 were not included in the more detailed analyses given their very low mortality rate (<1 death per 1,000 person years) and corresponding absolute number of deaths. Data from four new villages added to the site since 2007 were also not included in the analysis as they contributed minimal data to the study period.

### Outcome and explanatory variables

The dichotomous age-specific mortality outcomes were defined as follows:

- Infant – mortality within the first 365.25 days of life
- Children – mortality between 1 and 4 years of age
- Young to middle-age adults – mortality between 15 and 49 years of age
- Older adults – mortality between 50 and 64 years of age

Person time was defined as time (in years) contributed by an individual during the study period until right censoring (0) or death (1). The time to right censoring was set to either the date of permanent out-migration during the study period or as 31 December 2008 if the individual was present and alive. Demographics (gender, nationality), time period, season, maternal factors (former refugee status, age at pregnancy, death of mother during their off-springs infancy or childhood, education) and fertility factors (parity, birth intervals, sibling death), household factors (size mortality experience, household head demographics, socio-economic status based on household assets, food security), health seeking (distance to nearest health facility, antenatal clinic attendance), migration patterns, and household elevation (climatic proxy) were included as explanatory variables. Household socio-economic status (SES) was based on living conditions, assets and services including building materials of main dwelling, water and energy supply, ownership of modern appliances and livestock, and means of transport. These assets were used to construct an SES index using a multivariate statistical technique for categorical data, namely multiple correspondence analysis (MCA) (15).

### Risk factor analysis

A preliminary bivariate risk factor analysis was conducted to assess the relationship between mortality and each covariate. Covariates significant at the 10% level were then incorporated into the multivariable model. Given the inherent spatial and temporal correlation of longitudinal HDSS data, problems arise when using standard statistical methods as they assume independence of outcome measures (e.g. mortality). Objects in close proximity are often more alike, and common exposures (measured or unmeasured) may influence adult mortality similarly in households of the same geographical area, introducing spatial correlation in mortality outcomes. Including the spatial effect of proximity is important for an efficient estimation of parameters and prediction (16). Ignoring this correlation introduces bias in the risk factor analysis as the standard error of the covariates is underestimated, thereby overestimating the significance of the risk factors. Geostatistical models relax the assumption of independence and assume that spatial correlation is a function of distance between locations. They are highly parameterised models, and their full estimation has only become possible in the last decade by formulating them within a Bayesian framework (17) and estimating the parameters via Markov chain Monte Carlo (MCMC) simulation. With the development of MCMC methods and software such as WinBUGS (18), Bayesian approaches are being applied to the analysis of many social and health problems in addition to disease mapping and modelling or kriging (19). Thus, Bayesian geostatistical multivariable models are needed to analyse longitudinal data in order to address these problems.

Different analytical dataset structures were used for the various age groups. This dictated the corresponding modelling approach to examine the multivariable association between the significant covariates and age-specific mortality. For infants, a negative binomial model (selected due to over dispersion) was used with an offset of time in days contributed in the first year given their higher risk earlier on. For children (1–4 years), a monthly discrete time logistic or event history approach was used to track any changes of selected covariates in the given intervals. A monthly time interval was used as it was a better approximation of the risk than using a yearly interval. For the adult models, a continuous time to event or survival approach (Weibull parametric model) was adopted that split episodes of time for any relevant changes in selected covariates, for example, change of location or household. For a detailed comparison of the strengths and weaknesses of each analytical approach, please see Appendix 1. Details of the infant and child statistical models and selected results have been published previously (20, 21). However, the infant results in this paper include important additional variables (mother death due to HIV or non-HIV, breastfeeding) not used previously. A spatial random effect at the village level was included to take account of spatial correlation and was modelled using a multivariate Gaussian distribution with a covariance matrix expressed as a parametric function of the distance between pairs of village centroids points (17). Furthermore, an unstructured household-level random effect was included to take into account repeated household observations where time episodes were split to incorporate any time varying issues such as change of household physical location. MCMC simulation (22) was employed to estimate the model parameters. Detailed formulation of the models as well as the WinBUGS codes to implement each can be found in Appendices 2 and 3, respectively.

### Model assessment

Model comparison in STATA was based on the Akaike information criterion (AIC). The deviance information criterion (DIC) was used to assess the various Bayesian multivariable models (23). Both the AIC/DIC are a measure of the relative goodness of fit of a statistical model. Generally, the smaller the AIC/DIC, the better the model fit.

### Spatial analysis

The Kulldorff spatial scan statistic (24) was used to identify significant spatial clustering of mortality. Simulation-based Bayesian Poisson kriging (25) was also used to produce smoothed maps of all-cause mortality risk within the whole HDSS area. All-cause and cause-specific baseline models used included a constant and spatial random effect only. All identifying features (such as village centroids, boundaries) have been removed from the maps to ensure confidentiality and avoid stigmatisation of potentially high-risk villages. The HIV and tuberculosis mortality risk map is also not shown for the abovementioned reason. Model spatial estimates were exponentiated to give relative risk (RR). Risk maps were developed using a heat scale of the location specific RR prediction. Darker areas reflect increasingly higher RR, while increasingly lighter areas indicate lowering RR. A simple map showing potentially high-risk areas as a function of straight-line distance to nearest health facility was constructed using a circular buffer zone around health facilities based on significant cut-off found in the risk factor analysis.

### Software

Data were extracted from the Agincourt database using Microsoft SQL Server. The analysis was carried out in STATA version 10.0 SE (26) and WinBUGS (18). Risk maps were constructed in MapInfo Professional version 9.5.

## Results

### Demographic and mortality profile

The demographic and mortality profile of the study samples are provided in Table 1. Overall 9,035 deaths occurred during 1992–2008, based on 1,110,166 person-year time contributed, at an overall crude mortality rate of 8.1 per 1,000 person-years. The highest mortality rates occurred among infants followed by the older adult (50–64 years) age group (29 and 19 per 1,000 person-years, respectively). The mortality rate among children and younger adults (15–49 years) was similar at 5.7 and 6.9 per 1,000 person-years, respectively. Among infants 216 deaths occurred during the perinatal^1^ period and 251 in the neonatal^2^ period, that is, the majority occurred in the perinatal or early neonatal phase. The overall perinatal and neonatal mortality rates were 7.6 and 8.8 per 1,000 person-years, respectively. The lowest mortality rates were observed in the 5–14 year age group. Among adults (15–64 years), mortality rates showed a steady increase by 5-year grouping with a non-linear excess in the 30–34 and 35–39 age groups due to HIV/AIDS.

**Table 1:** Demographic profile of study sample by age group, 1992–2008

Characteristic	Infants (<1 years)	Children (1–4 years)	Children (5–14 years)	Adults (15–49 years)	Adults (50–64 years)	Overall^b^
Denominator (person-years)	28,470	116,729	311,387	549,030	70,864	1,110,166
Female (%)	16,030 (50.4)	20,838 (50.3)	34,770 (50.8)	99,994 (56.6)	4,062 (59.5)	576,680 (51.9)
South African (%)	20,382 (64.1)	25,848 (62.3)	43,870 (65.2)	66,926 (66.9)	4,292 (62.9)	117,448 (64.1)
Deaths (% of overall deaths)	826 (9.1)	669 (7.4)	289 (3.2)	3,798 (42.0)	1,337 (14.8)	9,035 (100)
Mean age at death (standard deviation)	126.2 days (112.7)	2.1 years (0.9)	10.0 years (2.9)	34.6 years (8.5)	57.3 years (4.5)	41.8 years (26.8)
Median age at death (interquartile range)	99 days (16–217)	1.9 years (1.4–2.6)	10.0 years (7.5–12.5)	34.8 years (28.4–41.5)	57.0 years (53.5–61.4)	40.7 years (24.2–63.8)
Mortality rate^a^	29.0	5.7	0.9	6.9	18.9	8.1

a Per 1,000 person-years.

b Includes 5–14 and 65+ age groups.

### Leading causes of death by age group, 1992–2008

The leading cause-of-death in all age groups (Table 2) was HIV/TB. Among children, the second most prominent cause-of-death was diarrhoea or malnutrition. Among younger adults (15–49 years), external cases of death, namely assault and transport accidents, featured as the second and third top causes-of-death, with lifestyle-related diseases following. In the older adult age group (50–64 years) following HIV/TB, chronic non-communicable diseases featured prominently.

**Table 2:** Top five causes-of-death by age group, 1992–2008

Rank	Infants (<1)	No. (percent)	Children (1–4)	No. (percent)	Adults (15–49)	No. (percent)	Adults (50–64)	No. (percent)
1	HIV/TB	132 (16.6)	HIV/TB	192 (29.5)	HIV/TB	1,545 (43)	HIV/TB	317 (24.7)
2	Diarrhoea or malnutrition	104 (13.1)	Diarrhoea or malnutrition	164 (25.2)	Assault	164 (4.6)	Vascular	159 (12.4)
3	ARI or pneumonia	103 (13)	External	39 (6)	Transport accident	134 (3.7)	Neoplasms	88 (6.9)
4	Perinatal condition^a^	93 (11.7)	ARI or pneumonia	27 (4.2)	Vascular	109 (3)	Digestive	54 (4.2)
5	Congenital	25 (3.1)	Congenital	13 (2)	Neoplasms	106 (2.9)	Suicide	35 (2.7)

a Based on ICD-10 main cause-of-death only, that is, P00–P96 (not inclusive of date of birth and date of death timing).

### Temporal trends in age-specific mortality

**Fig. 2:**
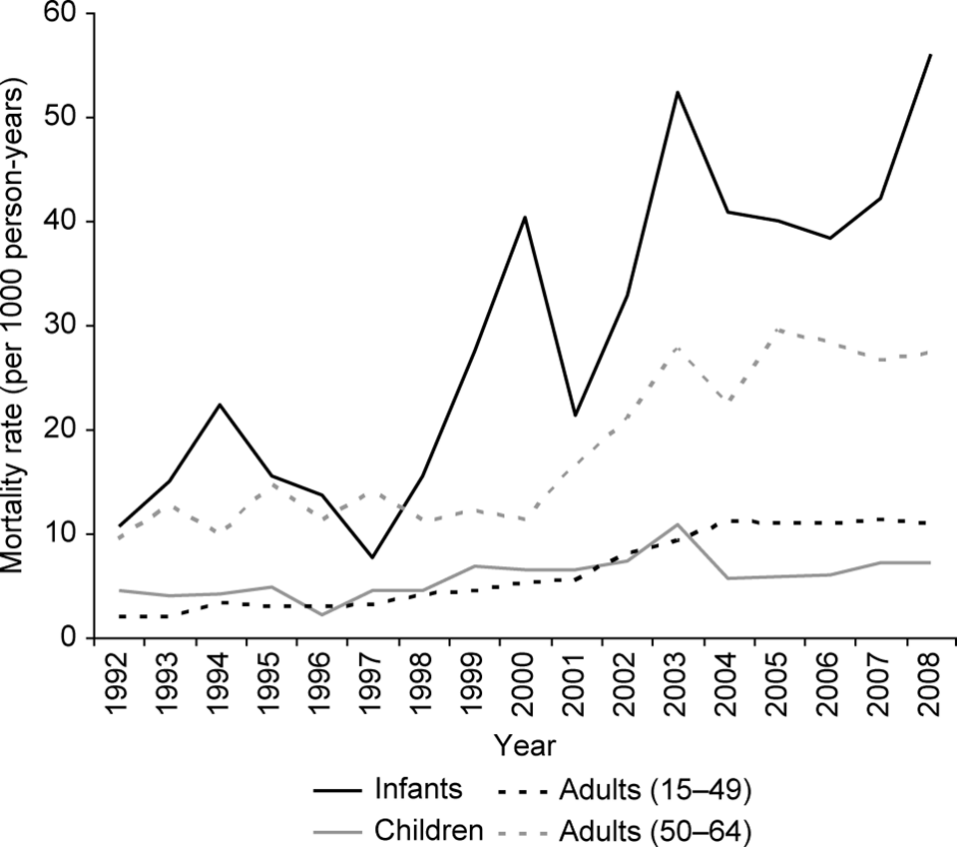
Age-specific mortality rates by year, 1992–2008.

A significant increase in all age group mortality rates was observed over the study period (Fig. 2), especially due to the impact of the HIV epidemic from the late 1990s to mid-2000s. All-cause mortality began to plateau around 2004 following rollout of the antiretroviral therapy (ART) programme in 2003 and reduction of HIV-related mortality. The temporal trend terms included in each multivariable model confirmed the significant increase in mortality across all age-groups over the study period both linearly and by period (Table 3).

**Table 3:** All-cause multivariable risk factor analyses for age-specific mortality using Bayesian modelling

	Infants (<1)	Children (1–4)^a^	Young adults (15–49)	Older adults (50–64)
	
Factors	IRR (95% BCI)^b^	OR (95% BCI)^b^	HR (95% BCI)^b^	HR (95% BCI)^c^
Temporal				
Year (continuous)	1.25 (1.07, 1.58)^c^	1.05 (1.04, 1.07)^f^	–	–
By period				
1992–1997	–	–	1	1
1998–2003	–	–	2.61 (2.42, 2.83)^c^	2.59 (2.16, 3.08)^c^
2004–2008	–	–	2.90 (2.52, 3.32)^c^	4.07 (2.85, 5.60)^c^
Proximate individual- and household-level determinants				
Increasing age in years	n/a	0.51 (0.46, 0.56)^b^	1.06 (1.06, 1.07)^c^	1.04 (1.02, 1.06)^c^
Winter season	n/a	1.33 (1.12, 1.57)^b^	–	–
Male gender	1.10 (0.83, 1.41)^c^	e	1.47 (1.38, 1.56)^c^	3.03 (2.64, 3.48)^c^
Mozambican (maternal for infants and children)	e	1.12 (0.90, 1.38)^b^	0.94 (0.87, 1.02)^c^	0.59 (0.50, 0.70)^c^
Maternal death (in first year for infants or 1–4 for children)				
Not due to HIV/TB	6.01 (3.18, 11.37)^c^	5.45 (2.99, 8.75)^b^	n/a	n/a
Due to HIV/TB	30.78 (12.13, 78.11)^c^	15.11 (8.39, 24.54)^b^	n/a	n/a
Migrant: ≥6 months outside site per year (maternal status for infants and children)	0.71 (0.48, 1.02)^c^	e	1.17 (1.09, 1.25)^c^	1.23 (1.04, 1.44)^c^
Paternal death (prior birth to within first year for infants or 1–4 for children)				
Not due to HIV/TB	e f	1.51 (0.75, 2.54)^b^^f^	n/a	n/a
Due to HIV/TB	e f	2.19 (0.95, 4.06)^b^^f^	n/a	n/a
Tertiary education (maternal for infants and children)	d	d	–	–
Secondary or higher for adult models	n/a	n/a	0.98 (0.91,1.05)^c^	0.56 (0.40, 0.77)^c^
Cumulative other household deaths	7.24 (5.70, 9.28)^c^	g	–	–
Number of other household deaths	g	g	–	–
None	g	g	1	e
1–4	g	g	1.17 (1.09, 1.25)^c^	e
5+	g	g	1.81 (1.39, 2.30)^c^	e
Household head:				
Male	e	0.78 (0.60, 1.02)^b^	0.53 (0.50, 0.57)^c^	0.40 (0.35, 0.45)^c^
Death	e f	d	4.52 (4.16, 4.91)^c^	6.74 (5.70, 7.92)^c^
≥40 years of age	1.44 (1.08, 1.90)^c^	e	0.62 (0.58, 0.66)^c^	0.15 (0.13, 0.17)^c^
Mozambican	e	1.71 (1.40, 2.09)^b^	e	e
Infant and child specific factors				
Parity	e	e	n/a	n/a
Death of previous sibling	1.56 (0.75, 2.90)^b^	1.63 (1.08, 2.45)^b^	n/a	n/a
Never breastfed	203.7 (113.9, 342.4)^c^^e^	e	n/a	n/a
Increasing birth weight	d	d	n/a	n/a
Other child born less than one year prior	8.07 (1.33, 28.02)^c^	g	n/a	n/a
Four or more children in the household	g	1.44 (1.13, 1.80)^b^	n/a	n/a
Socio-economic determinants				
Household SES (MCA) quintile for infant and child models; tertiale for adult models				
Most poor	e	g	1	1
Poor	e	g	0.76 (0.69, 0.84)^c^	0.78 (0.58, 1.05)^c^
Least poor	e	g	0.65 (0.58, 0.71)^c^	0.78 (0.58, 1.03)^c^
Unknown	e	g	1.48 (1.26, 1.71)^c^	1.01 (0.75, 1.35)^c^
Village level determinants				
Proportion of village deaths due HIV/TB (≥17%)^h^ (proxy for high prevalence)	–	–	1.92 (1.40, 2.69)^c^	e
Average migrant months per year per village individual	–	–	0.95 (0.87, 1.07)^c^	e
Environmental or geographic factors				
Distance to nearest health facility (>6 km)	e	e	5.34 (3.11, 9.98)^c^	1.20 (0.17, 8.63)^c^,^e^
Climatic proxy (elevation in metres)	e	e	0.998 (0.997, 0.998)^f^	0.998 (0.997, 0.999)^f^
Additional model parameters				
Individual unstructured variation (σ_u_^2^)	–	0.04 (0.01, 0.11)	0.11 (0.08, 0.14)	0.16 (0.10, 0.27)
Spatially structured variation (σ_ϕ_^2^)	0.08 (0.03, 0.23)	0.23 (0.10, 0.48)	2.21 (1.09, 4.11)	0.25 (0.11, 0.56)
Autoregressive structured temporal variation (σ_α_^2^)	0.60 (0.27, 1.57)	0.29 (0.12, 0.68)	–	–
Dispersion parameter (r) for negative binomial model	0.03 (0.025, 0.035)	–	–	–
Shape parameter (Weibull)	–	–	1.93 (1.86, 1.99)	1.92 (1.83, 2.01)
Deviance Information Criterion (DIC)	8,103	8,612	49,576	10,193

a Results for child model published previously in (45).

b Measure of association based on analytical data structure and model type for given age-group; incidence rate ratio (IRR), odds ratio (OR); hazard ratio (HR); BCI stands for Bayesian credibility interval (similar in interpretation to a conventional 95% confidence interval).

c Significant in bivariate analysis, estimate following multivariable adjustment.

d Significant (at 10% level) in bivariate analysis but substantial missing data so not included in final multivariable model.

e Not significant at 10% level in bivariate analysis.

f Small numbers (hence wide BCI's).

g Significant at bivariate level but not run in Bayesian multivariable framework due to co-linearity with another predictor.

h Median split.

### Major risk factors for age-specific mortality

Infants: maternal death in the infant's first year (especially due to HIV/TB), higher number of cumulative household deaths, no breastfeeding, and previous birth interval less than 1 year emerged as highly significant risk factors for all-cause infant mortality (Table 3). Mother being a migrant remained significantly protective. Male gender, increasing parity, and death of previous child were no longer significant risk factors following multivariable adjustment. No significant association was observed between infant mortality and household SES, increasing distance to nearest health facility and climate (using elevation as a proxy which corresponds to the rainfall gradient in the sub-district).

Children: maternal death between the child's first and fifth birthdays, particularly due to HIV/TB, was the most prominent risk factor from the multivariable analysis (Table 3), followed by father death due HIV/TB, four or more children aged less than 5 years living within the household, Mozambican origin of the mother, and winter season. Increasing age of the child remained highly protective. No significant association was observed between mortality risk and increased distance to nearest health facility. In contrast to infants, however, a significant and increasing trend of protective association was observed with increasing household SES based on the bivariate analysis.

Adults: The most prominent risks for 15–49 year mortality following multivariable adjustment were male gender, being a migrant, increasing number of other household deaths, household head death, and distance to nearest health facility (>6 km) (Table 3). Increasing wealth of household, household head being male, and older than 40 years were significant and prominent protective factors. Villages with a mortality proportion of HIV/TB above the median value remained at a significantly higher risk. Mozambican ethnicity and education were no longer significant after multivariable adjustment.

The most prominent risks for 50–64 year mortality following multivariable adjustment were male gender, being a migrant, and death of household head (Table 3). Households headed by older males again reduced older adult mortality risk. Mozambicans appeared to have significantly lower risk in this age group when compared to South Africans. In contrast to the findings for younger adults, following multivariable adjustment in the 50–64 year model, distance to nearest health facility (>6 km) and household SES were no longer significant risk factors.

**Fig. 3:**
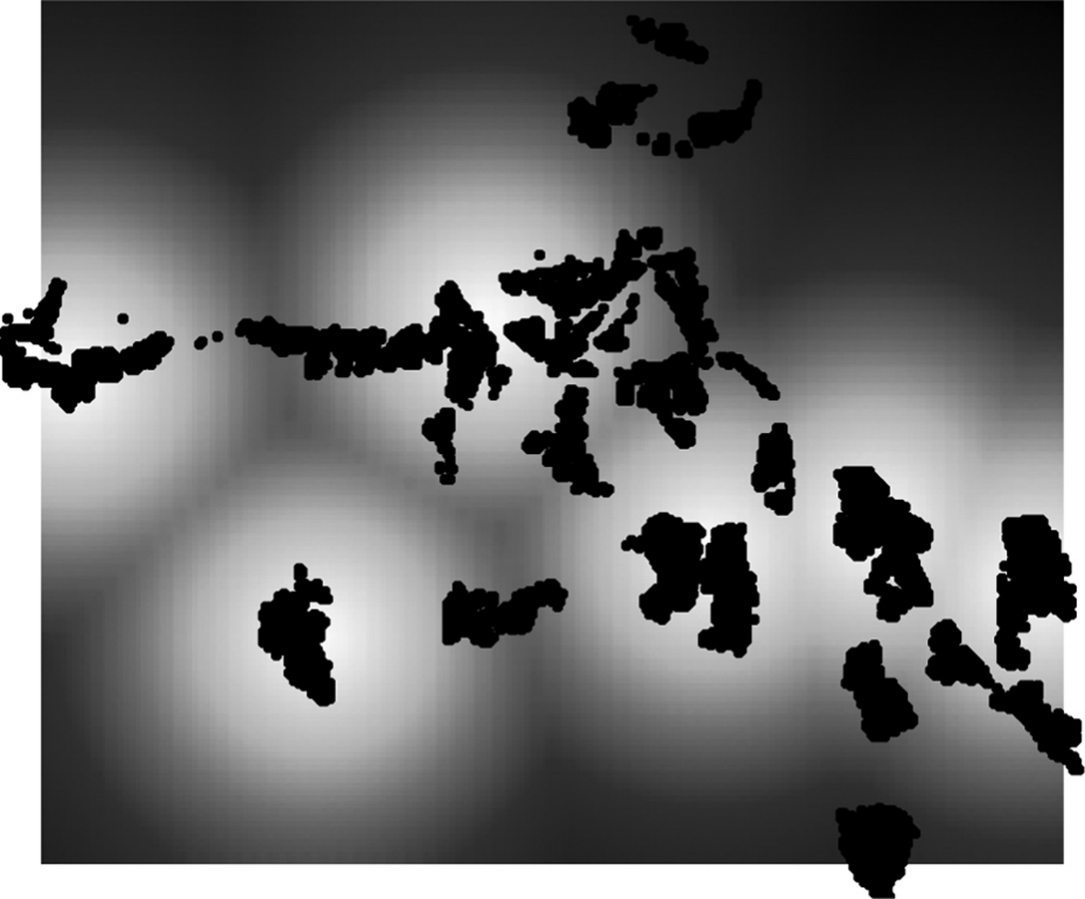
Geographic risk of adult mortality adjusting for distance to nearest health facility at prediction point using a univariate Bayesian spatial kriging model that includes distance to health facility as the risk factor (white and black scale represents lowest and highest risk, respectively).

Based on the risk prediction for straight-line distance to health facility, we can see that two villages in particular, one in the upper and the other in the lower south-east region, appear to have a higher mortality risk as a function of increased distance to the nearest local clinic in the Agincourt sub-district (Fig. 3). We also observe that there are other villages that appear to be far from the nearest health facility.

### Spatial distribution of age-specific mortality

**Fig. 4:**
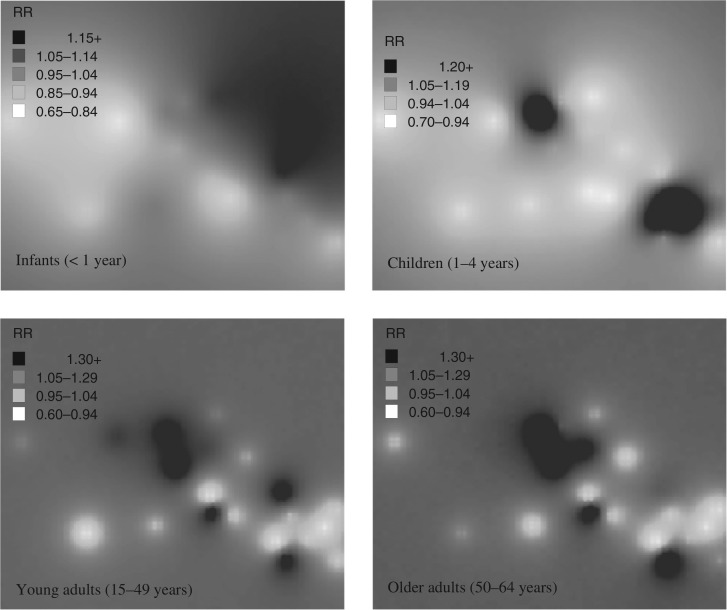
Maps of all-cause mortality risk by age group within the Agincourt sub-district based on baseline Poisson model without covariates.

Spatial risk estimates based on a Bayesian kriging model suggest a higher risk of infant mortality on the eastern border of the site while child mortality was concentred in two distinct foci: upper central and south-east corner of the sub-district. Five distinct foci of higher mortality in the 15–49 year age-group were observed using Bayesian kriging (Fig. 4). Three are in the central to upper central region of the site and two in the south east. These correlate to areas with higher risk of infectious disease mortality in this age group, largely HIV/TB. A very similar pattern was seen in the 50–64 year age-group when compared to 15–49 years though with one minor difference in that one village in the south-east was no longer at higher risk and one additional village in the upper central region emerged as high risk. Similarly, this distribution is largely driven by HIV/TB mortality. Higher non-communicable disease mortality risk was observed in one particular village in the upper central region of the site.

### Potential proximate reasons for the observed high-risk clusters

The high age-specific mortality clusters (Kulldorff spatial cluster scan statistic *p*<0.05), when compared to the lower mortality clusters, had significantly (*p*<0.05):

- more deaths due to HIV/TB and diarrhoea or malnutrition
- lower duration of breastfeeding
- higher number of deaths of previous children
- higher number of mothers dying of HIV/TB
- lower mean maternal education years
- higher number of cumulative household deaths
- younger household heads
- higher incidence of household heads dying
- more Mozambican household heads
- lower SES
- higher temporary migration rates
- lower proportion of individuals with secondary or higher level education.

## Discussion

This study has demonstrated the usefulness of advanced epidemiological modelling in assessing risk factors and producing smooth maps of mortality risk in a population.

Earlier work in South Africa and Agincourt has shown the profound impact of HIV/TB on mortality across most age groups (27–29), with higher than normal mortality rates evident. The results show that infectious diseases (particularly HIV/TB) were the most prominent cause-of-death over the study period and have largely contributed to the observed mortality trends. The levelling out of mortality round 2005 is possibly linked to the ART rollout, which began in South Africa round 2004. Current studies in this area are assessing the impact of ART rollout on mortality, as well as specific villages or areas where equity of access may be an issue. Mortality from non-communicable disease has also increased significantly in adults 30 years and older in the rural Agincourt sub-district (29) and has implications with regard to the epidemiological transition.

Results suggest that strong and significant space–time mortality disparities exist, even within a small geographic area. This distribution is being driven by a complex web of multilevel interacting factors that have likely increased communicable disease mortality (HIV) and non-communicable disease mortality (in the older age-group) in specific risk areas. According to the spatial analyses, the south-east and upper central regions of the site were consistently identified as high-risk areas for most age groups, thus indicating a definite non-random element to the mortality distribution in this rural sub-district. A strong geographical pattern of higher infectious disease mortality risk (particularly HIV/TB and diarrhoea/malnutrition) and former Mozambican settlements lying to the east of the site was also generally observed. Mozambican settlements in the south-east have generally been shown to have poorer access to water, sanitation, and waste disposal; in addition, they had fewer schools and poor quality of housing and were particularly isolated from public transport (30, 31).

Key individual and household level determinants have been confirmed and certain novel determinants have emerged. The results confirmed the importance of infant and child mortality risk factors such as maternal age, birth spacing, season, village and ethnic group (32, 33). Lack of breastfeeding in infancy and maternal death during infancy or childhood (1–4) were major risk factors as were a higher number of cumulative household deaths. Other reports indicate that infants who survive the death of the mother have a 10% chance or less of living past the age of 1 year (34). A complex interaction of higher HIV prevalence, large distance from nearest health facility, low SES, low education, high household mortality burden, and high migrancy rates (increased risk behaviour) appear to be driving communicable disease mortality, particularly HIV. Low SES and few employment opportunities locally have led to adults migrating externally for work. Migration patterns have been shown to influence HIV risk (35, 36), and temporary or labour migrants are more vulnerable to HIV than more settled populations. This has been shown in other African and southern African countries (37). One village in the upper central region appears to be at consistently higher risk across all age groups and has a significantly younger and highly mobile population, potentially engaging in higher risk behaviour with more time spent away as described by Collinson (31). Health education messages are also needed that focus on high-risk sexual behaviour that increases risk for HIV infection, and its consequences. Death of household members also appeared to be a significant risk in all age-groups. Previous research indicates that HIV has arguably had the greatest impact at the household level in terms of dissolution and reduced economic status (38).

There are several studies relating geographical access to use of health facilities. As one would expect, members of communities that are more distant use the facilities less than those that live nearer, but this does not necessarily translate into increased mortality risk (39). In this study, we did not observe significant excess risk associated with increased distance to nearest health facility among infants and children, and this has been demonstrated previously in this setting (20, 21). Conversely, larger distance from the nearest health facility had a significantly higher risk associated with adult (15–49 years) mortality. This has been shown in a previous study on adult mortality in China (40).

A limitation of the study is the potential to miss infant deaths, particularly neonatal deaths, which would underestimate the overall infant mortality burden. However, infant death ascertainment has improved in the study site, especially towards the end of the study period (41). Determination of cause-of-death through VA is more problematic for diseases that have less specific symptoms such as HIV/AIDS (42). Thus it is likely that the HIV burden may have been underestimated. However, previous validation studies of the VA in Agincourt HDSS have shown that it performs well in this high HIV prevalence setting (43). Levels of stigma associated with HIV are high in South Africa particularly prior to the introduction of HAART and may have also contributed to this underestimation.

## Conclusion

This work has contributed through the testing, refinement, and application of various advanced spatial–temporal analyses and statistical modelling of risk factors to large longitudinal cohorts such as an HDSS. The novel application of methodologies in public health contributes to our understanding of factors related to mortality and how to quantify them accurately for correlated geostatistical and longitudinal data. This study also contributes to the development of public health interventions by targeting clusters of adverse health outcomes that appear to aggregate geographically and in time as well as the tracking and targeting of other emerging (or re-emerging) communicable diseases that are compromising achievements made in developing countries (44). In particular, space–time modelling and mapping can be an effective tool in public health by showing and monitoring diffusion patterns of communicable diseases and in searching for infectious agents. The identification of disparities in the distribution of mortality and related risk factors in space and time, can guide effective policy interventions and programmes. The methods developed, assessed, and used in this thesis contribute to our understanding of risk factor modelling of large correlated longitudinal data.

This study should be regarded as a first step in prioritising specific areas for follow-up public health efforts and evaluating their impact in this rural setting. Targeting prevention of HIV/TB and antiretroviral rollout in significant child and adult mortality clusters and ensuring maternal survival appear key to improving infant and child mortality rates. Further spatial assessment of antiretroviral therapy (ART) rollout that started in this area in 2007 as well as identifying any villages or areas not accessing ART equitably is also critical. The provision of adequate water and sanitation is needed in the mortality clusters particularly in the south-east where diarrhoeal mortality appears high.

## Footnotes

1 Perinatal period: last period of gestation up to first 7 days of life.

## Footnotes

2 Neonatal period: within the first 28 days of life.
